# Supplementary material for: A versatile enhanced freeze-substitution protocol for volume electron microscopy
Source: Front Cell Dev Biol. 2022 Aug 8;10:933376. doi: 10.3389/fcell.2022.933376 (PMC9393620; doi:10.3389/fcell.2022.933376)
Supplement: Supplementary file 3 [file DataSheet1.pdf]

SUPPLEMENTARY DATA

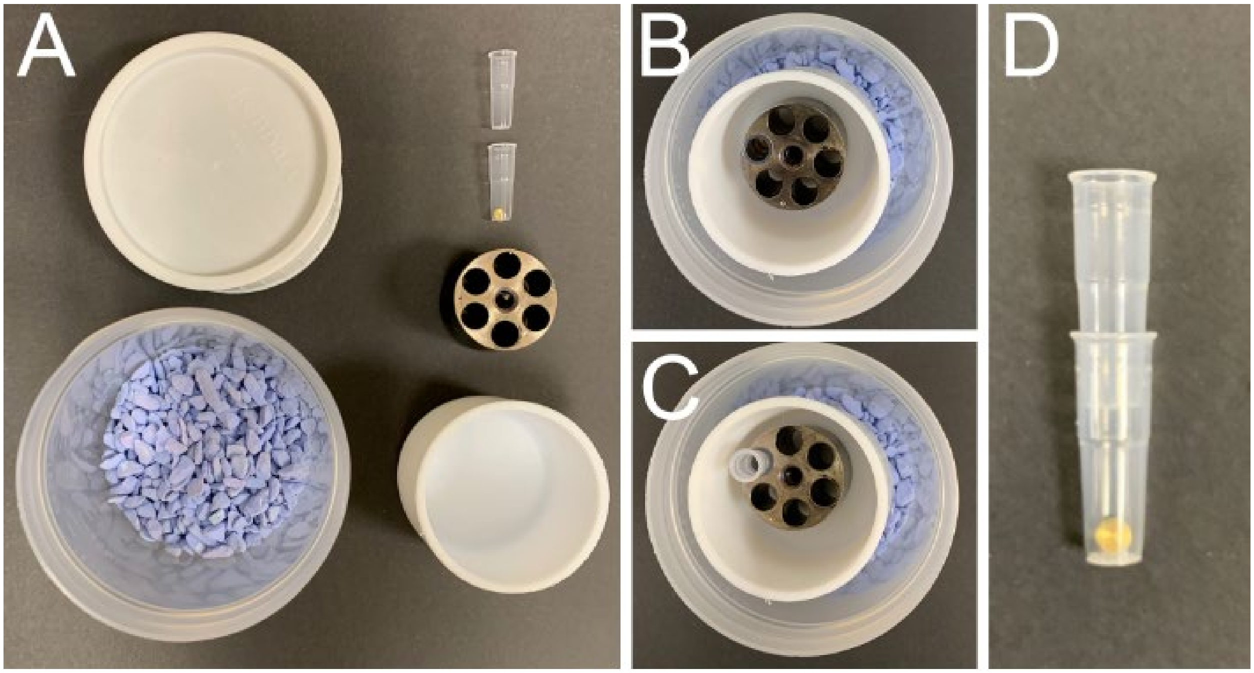

Supplementary Data 1. FSaqOTO mPrep capsules and containers used for freeze-substitution of plant materials at room temperature and shown here without reagents. **A.** Top view (start upper left and follow counterclockwise) of specimen cup lid, specimen cup with Drierite^{TM} desiccant, Teflon^{TM} substitution fluid container, mPrep capsule CPD holder, mPrep capsule bottom with HPF planchette (and specimen) inside, and the second mPrep/s capsule used as a top. **B.** Top view of assembled specimen cup with mPrep CPD holder within Teflon^{TM} substitution fluid container in specimen cup. **C.** Same as B, but with a bottom mPrep/s capsule to hold HPF planchette for processing. **D.** Configuration of final mPrep capsule assembly to entrap the sample with HPF planchette (and specimen) between two mPrep/s capsules.

Sample Preparation Steps

Sample	Freezing Method	FS Fix	FS Solvent	Warm-up	Polar Solvent to Aqeous Transition	Hua Protocol	KFeCN	TCH	OsO_4	Pb aspartate	Uac	Dehydration	Resin
H. vulgare Root	Leics EM Ice HPF	2% $Os0_4$	97% acetone +3% water	Manual	to 2% Os04 in 0.1M Na cacodylate	Yes	2.5% in water	1% in water	1% in water	50°C	1% in water 4°C/50°C	Acetone/Propylene Oxide	Quetol 651 hard formulation
H. vulgare Anther	Leics EM Ice HPF	2% $Os0_4$	97% acetone +3% water	Manual	to 2% Os04 in 0.1M Na cacodylate	Yes	2.5% in water	1% in water	1% in water	50°C	1% in water 4°C/50°C	Acetone/Propylene Oxide	Quetol 651 hard formulation
C. elegans	Leics EM Ice HPF	1% $Os0_4$	97% acetone +3% water	QFS	to 2% Os04 in 0.1M Na cacodylate	Yes	2.5% in water	1% in water	1% in water	50°C	1% in water 4°C/50°C	Acetone	PolyBed hard formulation
C. elegans	Leics EM Ice HPF	2% $Os0_4$	99% acetone + 1% water	AFS	None	No	X	X	X	X	1% in acetone room temp	Acetone	PolyBed hard formulation
S. cerevisiae	Leics EM Ice HPF	1% $Os0_4$ + 1% UAc	90% acetone + 5% methanol + 5% water	QFS	to 2% Os04 in 0.1M Na cacodylate	Yes	2.5% in water	1% in water	1% in water	50°C	1% in water 4°C/50°C	Acetone	Quetol 651 hard formulation
S. cerevisiae	Leics EM Ice HPF	1% $Os0_4$ + 1%glut	99% acetone + 1% water	AFS	None	No	X	X	X	X		Acetone	PolyBed hard formulation

AFS - Automated Freeze-Substitution, QFS = Quick Freeze-Substitution, KeFeCN = potassium ferocyanide, TCH = thiocarbohydrazide, OsO_4 = osmium tetroxide, Pb = lead, UAc = uranyl acetate

Supplementary Data 2. Sample preparation steps from freeze-substitution to resin.

Samples and Image Conditions

Figure	Sample Type	Freeze-subsitution Protocol	Imaging Platform	vEM Platform/Mode	FCC N_2 Settings (%)	X-Y resolution	Z-Resolution (slice thickness)	Image Pixel Resolution	Voltage (kV)	Beam Current	Dwell/Image-Time
2A	Barley Root	Manual FSaqOTO	ZEISS GeminisSEM300	Gatan 3-View 2XP w FCC	10	130nm	NA	3000x4000	5	1pA	3µs
2B	Barley Root	Manual FSaqOTO	ZEISS GeminisSEM300	Gatan 3-View 2XP w FCC	10	5nm	50nm	10000x10000	5	1pA	3µs
2C	Barley Root	Manual FSaqOTO	ZEISS GeminisSEM300	Gatan 3-View 2XP w FCC	10	5nm	50nm	10000x10000	5	1pA	3µs
2D	Barley Root	Manual FSaqOTO	ZEISS GeminisSEM300	Gatan 3-View 2XP w FCC	10	5nm	50nm	10000x10000	5	1pA	3µs
3A	Barley Anther	Manual FSaqOTO	ZEISS GeminisSEM300	Gatan 3-View 2XP w FCC	25	130nm	NA	4000x4000	5	1pA	4µs
3B	Barley Anther	Manual FSaqOTO	ZEISS GeminisSEM300	Gatan 3-View 2XP w FCC	35	5nm	50nm	10000x10000	5	1pA	3µs
3C	Barley Anther	Manual FSaqOTO	ZEISS GeminisSEM300	Gatan 3-View 2XP w FCC	35	5nm	50nm	10000x10000	5	1pA	3µs
3D	Barley Anther	Manual FSaqOTO	ZEISS GeminisSEM300	Gatan 3-View 2XP w FCC	35	5nm	50nm	10000x10000	5	1pA	3µs
3E	Barley Anther	Manual FSaqOTO	ZEISS GeminisSEM300	Gatan 3-View 2XP w FCC	35	5nm	50nm	10000x10000	5	1pA	3µs
Suppl_Video 1	Barley Anther	Manual FSaqOTO	ZEISS GeminisSEM300	Gatan 3-View 2XP w FCC	35	5nm	50nm	10000x10000	5	1pA	3µs
Suppl_Video 2	Yeast	QFSaqOTO	ZEISS CrossBeam550	Capella FIBSEM ATLAS 5	NA	5 nm	15nm	2661x626	1.5	1.5µA	3µs/1 line average
4A	Nematode	QFSaqOTO	ZEISS CrossBeam550	Brightfield STEM	NA	10nm	70nm	2048x2048	30	600pA	3µs/1 line average
4B	Nematode	AFSsolvOTO	ZEISS CrossBeam550	Brightfield STEM	NA	10nm	70nm	2048x2048	30	600pA	3µs/1 line average
4C	Nematode	QFSaqOTO	ZEISS CrossBeam550	Brightfield STEM	NA	3nm	70nm	2048x2048	30	600pA	3µs/1 line average
4D	Nematode	AFSsolvOTO	ZEISS CrossBeam550	Brightfield STEM	NA	3nm	70nm	2048x2048	30	600pA	3µs/1 line average
5A	Yeast	QFSaqOTO	ZEISS CrossBeam550	Brightfield STEM	NA	10nm	70nm	2048x2048	30	600pA	3µs/1 line average
5B	Yeast	AFSsolvOTO	ZEISS CrossBeam550	Brightfield STEM	NA	10nm	70nm	2048x2048	30	600pA	3µs/1 line average
5C	Yeast	QFSaqOTO	Hitachi H7650	TEM	NA	2nm	70nm	2048x2048	80		
5D	Yeast	AFSsolvOTO	Hitachi H7650	TEM	NA	2nm	70nm	2048x2048	80		

Supplementary Data 3. Sample and image conditions table providing major acquisition parameters (SBF-SEM, FIB-SEM, STEM and TEM) for all included images of barley root and anther (*Hordeum vulgare ssp. vulgare*), yeast (*S. cerevisiae*) and nematode (*C. elegans*) samples.

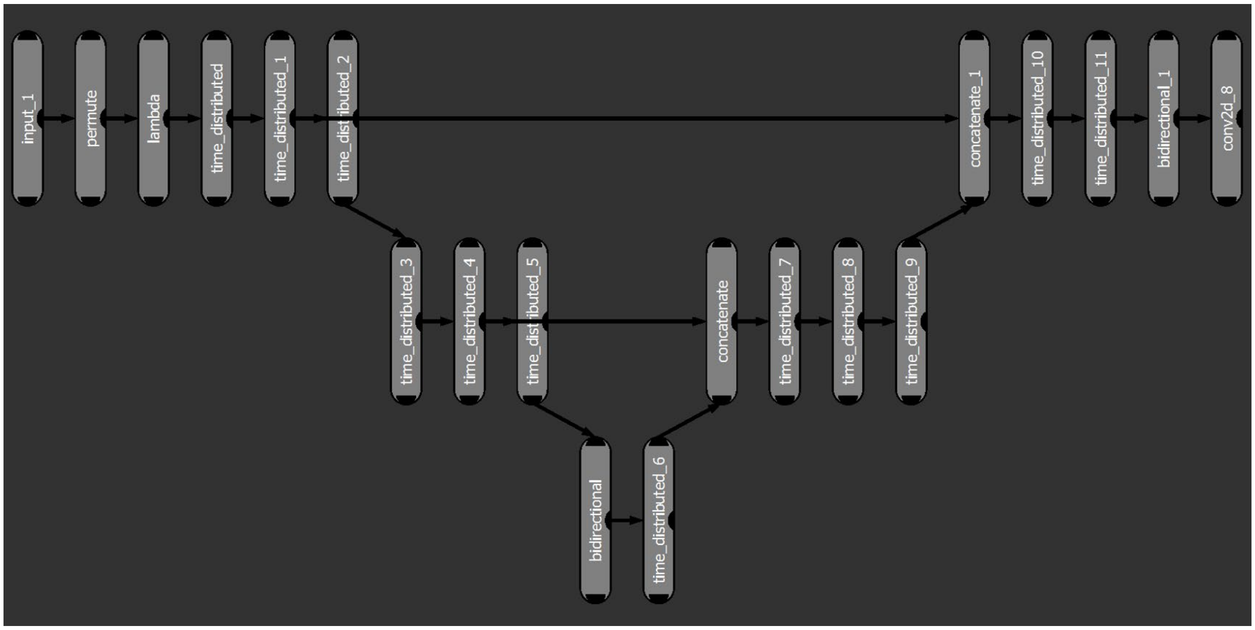

Supplementary Data 4. 3D Sensor Deep learning model parameters used with ORS Dragonfly segmentation of barley root (**Fig 2E**).

Supplementary Data Video 1. SBF-SEM stack of high-pressure frozen barley root prepared by FSaqOTO.

Supplementary Data Video 2. FIB-SEM stack of high-pressure frozen yeast (*S. cerevisiae*) by FSaqOTO.
